# Behavior of Polymer Electrode PEDOT:PSS/Graphene on Flexible Substrate for Wearable Biosensor at Different Loading Modes

**DOI:** 10.3390/nano14161357

**Published:** 2024-08-17

**Authors:** Mariya Aleksandrova, Valentin Mateev, Ivo Iliev

**Affiliations:** 1 Department of Microelectronics, Technical University of Sofia, 1000 Sofia, Bulgaria; 2 Department of Electrical Apparatus, Technical University of Sofia, 1000 Sofia, Bulgaria; vmateev@tu-sofia.bg; 3 Department of Electronics, Technical University of Sofia, 1000 Sofia, Bulgaria; izi@tu-sofia.bg

**Keywords:** polymer electrode, PEDOT:PSS/graphene, flexible electronics, wearable biosensors

## Abstract

In recent years, flexible and wearable biosensor technologies have gained significant attention due to their potential to revolutionize healthcare monitoring. Among the various components involved in these biosensors, the electrode material plays a crucial role in ensuring accurate and reliable detection. In this regard, polymer electrodes, such as Poly(3,4 ethylenedioxythiophene): poly(styrenesulfonate), combined with graphene (PEDOT:PSS/graphene), have emerged as promising candidates due to their unique mechanical properties and excellent electrical conductivity. Understanding the mechanical behavior of these polymer electrodes on flexible substrates is essential to ensure the stability and durability of wearable biosensors. In this paper, PEDOT:PSS/graphene composite was spray-coated on flexible substrates at different growth conditions to explore the effect of the deposition parameters and mode of mechanical loading (longitudinal or transversal) on the electrical and mechanical behavior of the fabricated samples. It was found that the coating grown at lower temperatures and higher spraying pressure exhibited stable behavior no matter the applied stress type.

## 1. Introduction

The increasing demand for wearable biosensors has led to extensive research on efficient and comfortable healthcare monitoring devices [1,2,3,4]. These biosensors offer the potential for continuous and real-time monitoring of various physiological parameters, making them invaluable tools for personalized healthcare and disease management. One critical aspect of wearable biosensors is the choice of electrode material, as it influences the performance and durability of the device. Traditional metal-based electrodes face limitations in flexibility and stretchability, making them less suitable for wearable applications [5,6]. In contrast, polymer-based electrodes have opened up new avenues for developing flexible and stretchable biosensors [7,8,9].

Among the various polymer electrode materials, PEDOT:PSS stands out due to its excellent electrical conductivity, transparency, and ease of fabrication. PEDOT:PSS can be easily solution-processed and patterned onto flexible substrates, making it compatible with large-scale manufacturing processes. In recent years, the conductivity of the PEDOT:PSS films has been enhanced by a variety of approaches to make them suitable for electrodes of different electronic devices. Some include multilayer stacks with intermediate ultrathin (few nanometers) silver coating or silver grids formed by lithographic patterning [10]. Another approach has been the introduction of gold or copper nanoparticles [11,12]. Some of the popular treatments for improving PEDOT:PSS conductivity are solvent doping with ethylene glycol (EG), polyethylene glycol (PEG), diethylene glycol, dimethyl sulfoxide (DMSO), dimethyl sulfate, tetrahydrofuran (THF), and N,N-dimethyl formamide (DMF); treatment with ionic liquids such as 2-methylimidazolium chloride; and 2-methylimidazolium hydrogen sulfate or treatment with acid, such as boric or sulfuric acid [13,14,15,16,17]. Composites with fillers like graphene or graphene oxide can also be found in the literature [18].

Furthermore, incorporating graphene into PEDOT:PSS films enhances the electrical properties due to the outstanding conductivity of graphene. While the electrical properties of PEDOT:PSS/graphene films have been extensively studied, their mechanical behavior and long-term stability on flexible substrates are still an area of active investigation. The flexibility and stretchability of the electrode material are critical for wearable biosensors, as they need to come into contact with the curved surfaces of the human body without compromising their electrical performance.

Spray deposition offers advantages such as simplicity, scalability, and the ability to produce uniform and conformal coatings, making it suitable for large-scale manufacturing of wearable biosensors. By incorporating spray deposition into the fabrication process, this study addresses the practicality and manufacturing aspects of employing the PEDOT:PSS/graphene electrode. Process parameters involved in spray deposition, such as spraying temperature, spraying pressure, and number of scans, can affect the mechanical properties of the PEDOT:PSS/graphene electrode. Investigating the relationship between spray deposition parameters and the resulting mechanical behavior of the electrode provides insights into optimizing the deposition process to enhance the flexibility and durability of the electrode. This knowledge can contribute to the development of robust and reliable wearable biosensors. While other fabrication techniques, such as spin coating or inkjet printing, have been explored for electrode deposition, the use of spray deposition in this study offers unique advantages [19,20,21].

Typically, wearable electrodes are designed to withstand tensile stresses while maintaining their functionality and comfort. Tensile stress can arise from movements like stretching, bending, and twisting of the body, or external mechanical forces applied to the electrode [22]. The realistic tensile stress that a wearable electrode can experience depends on several factors, including the specific design and materials used in the electrode, the area of the electrode in contact with the skin, and the nature of the human physical activity. To ensure the electrode’s reliability and prevent discomfort or damage to the skin, it is common for wearable electrodes to be designed to withstand tensile stresses in the range of a few kilopascals (kPa) to tens of kilopascals, which is in the range of 50–100 g-force/cm^2^ [23]. It is important to note that individual activities and movements can generate different tensile stress levels. For example, high-impact physical activities or extreme body movements may exert higher tensile forces than regular daily activities. Therefore, when designing wearable electrodes, it is essential to consider a safety margin and ensure that the electrodes can withstand a reasonable range of tensile stresses without compromising their performance or causing discomfort to the wearer. The specific loading method can influence how the sample deforms in longitudinal (along the length) or transverse (perpendicular to the length) distribution of deformation. For example, if a tensile or compressive load is applied along the length of a rectangular sample, the deformation will primarily occur in the longitudinal direction [24]. It means that the sample will elongate or compress along its length, while its width and thickness experience minimal change. On the other hand, if a bending or twisting load is applied to a rectangular sample, the deformation will involve both longitudinal and transverse components. Bending or twisting can cause bending and shear stresses, resulting in deformation and strain in the longitudinal and transverse directions [25].

In this study, PEDOT:PSS/graphene on PET substrate was directly used instead of the layered structure involving ITO/PET, thus trying to investigate the possibility of avoiding the inclusion of ITO and achieving simplicity and cost-effectiveness. Directly applying PEDOT:PSS/graphene on PET substrate, having competitive conductive properties, eliminates the need for an additional layer of ITO. At the same time, PEDOT:PSS/graphene is known for its flexibility, which aligns well with the inherent mechanical behavior of PET substrates. By eliminating the rigid ITO layer, the overall flexibility and conformability of the sensor can be enhanced, making it more suitable for wearable applications. The direct application can enhance the bond between the conductive organic coating and the substrate, potentially reducing the risk of delamination or cracking during use. Understanding the behavior of the polymer electrodes at different loading modes is crucial for ensuring the reliability, durability, and user comfort of wearable biosensors. The exploration of PEDOT:PSS/graphene materials and their mechanical properties will pave the way for developing optimized electrodes for wearable biosensors capable of delivering accurate and reliable health monitoring in real-world applications. The combination of PEDOT:PSS and graphene into a single layer offers a compelling synergy between the excellent electrical conductivity of graphene and the desirable properties of PEDOT:PSS, such as ease of fabrication. This unique combination sets the study apart from previous research that has focused on other electrode materials. Although the electrical properties of PEDOT:PSS/graphene films have been extensively studied, the behavior of these electrodes on flexible substrates at different loading modes remains an area of active investigation. The limited literature on this specific topic indicates that this study fills a critical gap in knowledge.

This paper aims to study of the mechanical behavior of polymer electrode PEDOT:PSS/graphene on flexible substrates for wearable biosensors. The analysis and characterization techniques used to evaluate the mechanical properties, including tensile and bending tests, will be discussed. Additionally, the influence of various fabrication parameters, such as film thickness, surface roughness, and deposition conditions, on the mechanical performance of these electrodes will be explored.

## 2. Materials and Methods

**Figure 1:**
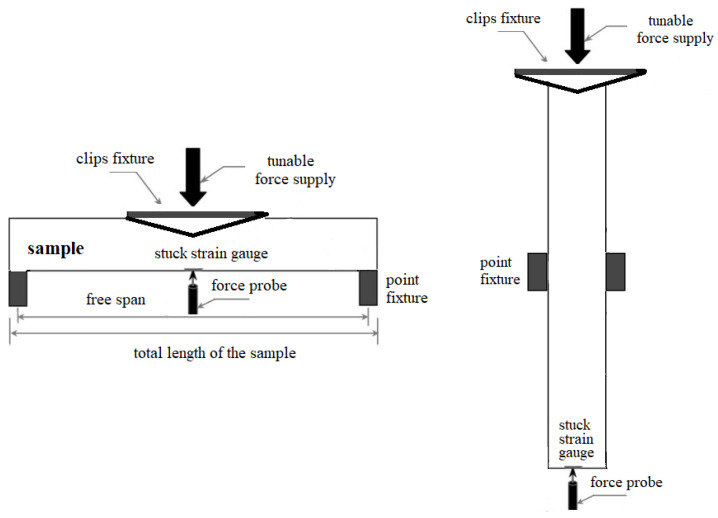
Illustration of the sample subjected to transversal load (**left**) and longitudinal load (**right**).

The flexible rectangular PET substrates with a thickness of 270 µm undergo cleaning using an isopropyl alcohol bath in an ultrasonic cleaner for 3 min. The hybrid ink of PEDOT:PSS/graphene (Sigma-Aldrich, Burlington, MA, USA) at 1 mg/mL concentration was mixed in DMF solvent. A spray coating setup, equipped with a 1 µm atomization nozzle and hot plate, was used for sample fabrication. Different numbers of passes were used to control the film’s thickness. The substrate temperature varied between 90 and 105 °C to regulate the liquid flow rate and the film’s uniformity. As it is expected that possible thermal instability can come from the PEDOT:PSS polymer in this composite, its thermal properties have been considered during the setting of the spray deposition temperature, considering results reported in [26]. In this research, 120 °C has been pointed out as a critical temperature for weight loss of the material. With a maximum of 105 °C for the present spraying experiment, there is sufficient thermal budget to avoid any structural transformations in the material that could potentially cause electrical and/or mechanical instability. Moreover, this study reports morphology and conductivity changes in PEDOT:PSS for thermal heating/cooling cycles after four scans with the magnitude of the temperatures 200 °C ÷ −100 °C. These temperature cycles are not applicable to our case because, like a future constituent of a wearable sensor, the PEDOT:PSS/graphene film will experience temperature differences in the range between the body temperature and the ambient temperature. Table 1 outlines the specific deposition conditions while maintaining a constant pulverization pressure of 2 bar and 3.5 bar and a nozzle-to-substrate distance of 12 cm. Before coating, the substrates were exposed to ultraviolet light for 10 min. (250 W, 365 nm) to enhance the wetting conditions and hydrophilicity of the PET surface. During deposition, there was a 30 s interval between sprayings to remove residual solvent and prevent solution leakage. Figure 1 illustrates the test method, which is based on the conditions of the standard ISO 527-3 [27] related to tensile properties of films and sheets, adapted to the concrete case study.

Cyclic bending tests (tension/compression) were conducted on a lab-made beam-shaped tester with an equivalent mass loading of 110 g-force/cm^2^, and the durability of the films was studied by sheet resistance measurement (Van der Pauw method realized by four-point prober FPP 5000 Veeco (Plainview, NY, USA) to monitor if deviation from the initial sheet resistance appears. Clamps, specifically designed for holding rectangular samples, were used to hold them at one end while applying a twisting moment at the other. This attachment induces torsional stress within the sample. The cyclic mass-loaded samples were subjected to atomic force microscopy (FlexAFM, Nanosurf, Liestal, Switzerland) analysis to measure the coating uniformity, average roughness, coverage integrity, and defects after loading and their dependence on the deposition conditions. An AFM scan was not conducted for the sample with the thickness of 3 µm due to the sharp increase in the resistance out of the suitable range for electrode purposes. This analysis complements the mechanical test results and helps to identify the structural factors affecting the behavior. Electrical impedance spectroscopy (Hioki IM3570, Nagano, Japan) was applied for the admittance measurement of the samples to monitor the stability and degradation of the bulk of PEDOT:PSS/graphene layers.

## 3. Results

**Figure 2:**
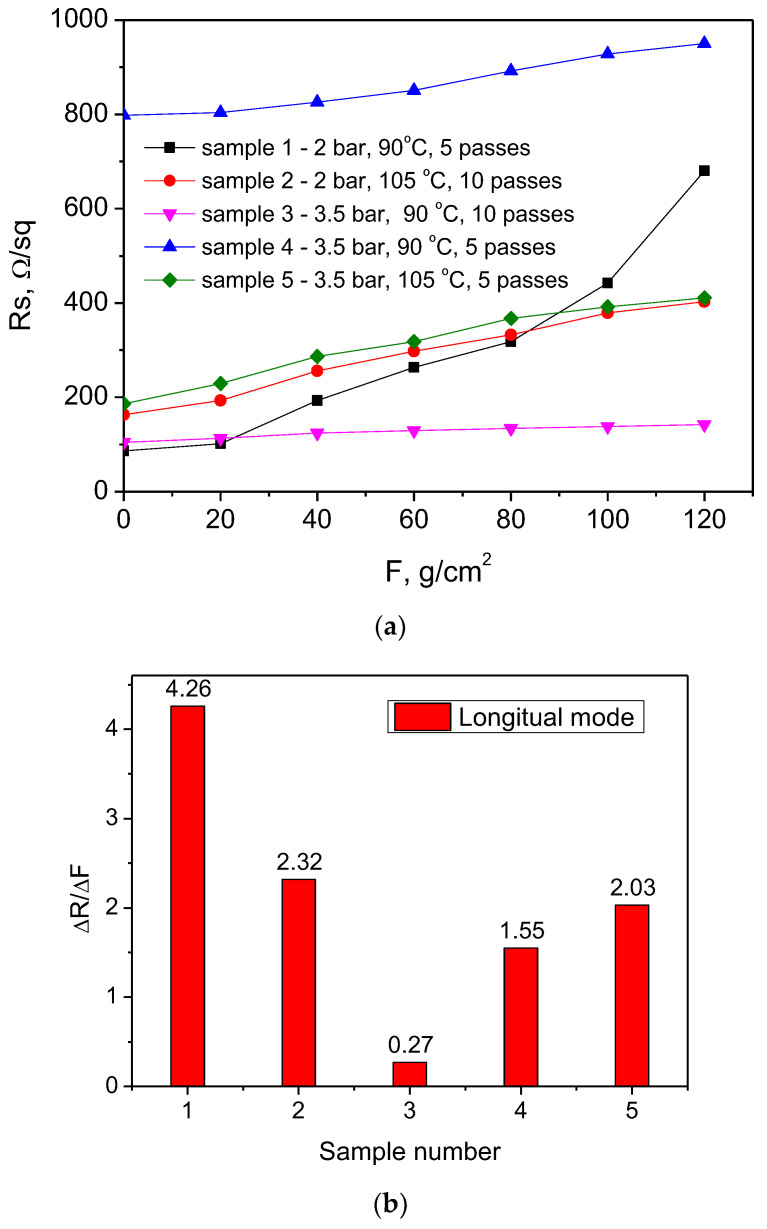
Sheet resistance variation (**a**) and sheet resistance degradation rate (**b**) for PEDOT:PSS/graphene films spray-coated at different conditions and subjected to longitudinal loading (denoted by L below).

The samples were subjected to longitudinal and transversal loading to investigate the effect of the mechanical loading type on the electrical characteristics and morphological features of the coatings grown at different spraying conditions. The results for the sheet resistance variation vs. applied force and degradation rates for all studied samples at longitudinal loading are shown in Figure 2a,b.

**Figure 3:**
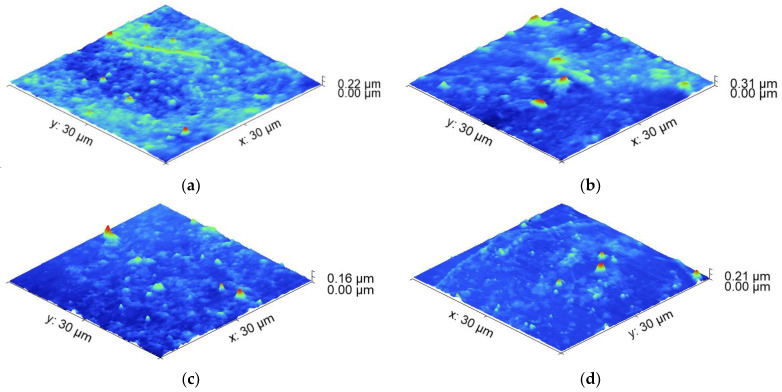
AFM 3D topography of the PEDOT:PSS/graphene coatings sprayed at different conditions and subjected to longitudinal loading for the samples with a thickness of (**a**) 3.1 µm; (**b**) 2.5 µm; (**c**) 1.75 µm; (**d**) 1.25 µm.

Sample 3L sprayed at 3.5 bar (higher pressure), ten passes, and 90 °C (lower temperature) exhibits the most stable resistance under longitudinal loading. Its resistance change was 0.27 Ω/sq per gram/cm^2^ load, and its initial resistance (~100 Ω/sq) was low enough to serve as an electrode. Sample 4, spray-coated at the same conditions but at five passes, reducing its thickness from 2.5 µm to 1.75 µm, is the next relatively stable at loading. However, its starting resistance is about 800 Ω/sq, which is too high to serve as an electrode. Sample 1L, sprayed at 90 °C, 2 bar and five passes demonstrated the most unstable behavior with the greatest variation in the sheet resistance and rate of its degradation of 4.26 Ω/sq per gram/cm^2^. Sample 5L and Sample 2L are similar in terms of resistance change. They were obtained at the same temperatures (105 °C) but at different pressures: sample 2L at a lower pressure of 2 bar and sample 5 at a higher pressure of 3.5 bar. It was suggested that the combination of higher pressure and a greater number of passes contributed to the stability and low resistance change. The lower temperature likely helped to maintain the stability of the resistance at longitudinal loading. The results also suggest that the film’s thickness, which is affected by the number of passes, plays a crucial role in the electrical performance of the samples. While pressure variation does not have a significant effect on the resistance change, it might have influenced other mechanical properties such as crack formation, which affects the electrical behavior. For a better understanding of the connection between the deposition conditions and resistance change after loading, AFM scans of the samples were conducted and the surface roughness was measured (Figure 3a–d).

Table 2 presents the relation between the resulting surface parameters of the longitudinal loaded films extracted from the AFM data and the spraying deposition conditions.

The comparison of the surface roughness of the sprayed films before and after longitudinal loading supports the results obtained from the sheet resistance measurement. The most unstable coating in sheet resistance variation (90 °C, five passes, 2 bar—lower deposition temperature, a smaller number of scans, lower spraying pressure) demonstrated a more than double increase in the RMS roughness from 16.7 to 36.41 nm after loading. The coating with the most stable sheet resistance (90 °C, 10 passes, 3.5 bar—lower deposition temperature, greater number of scans, higher spraying pressure) exhibited a small increase in the RMS roughness of 2.78 nm. The samples showing intermediate sheet resistance change of 2.03 Ω/sq per gram/cm^2^ also have an intermediate value of the RMS roughness increase with approximately 4.4 nm.

**Figure 4:**
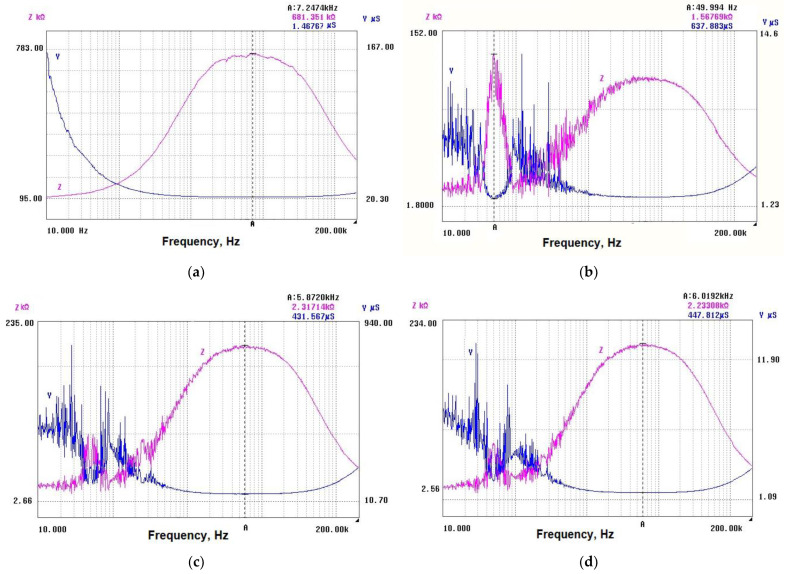
Admittance and impedance of samples with different thicknesses, subjected to longitude loading: (**a**) 3.1 µm; (**b**) 2.5 µm; (**c**) 1.75 µm; (**d**) 1.25 µm.

Admittance values for the L-series of samples are shown in Figure 4a–d. As is expected, a relatively high admittance of ~637 µS was measured for sample 3L due to its stable resistance behavior and low initial resistance. Sample 4L demonstrates lower admittance of 431 µS, probably due to the higher initial resistance, but it also shows a slower rate of change in admittance over time. Sample 1L may be expected to have a lower admittance due to its unstable behavior and high resistance change. The measured value for sample 2L was 1.46 µS. Samples 5L and 2L showed similar resistance changes and were obtained at the same temperature. Sample 5L was obtained at a higher pressure and demonstrated stability. Given the stability of these samples, it might be expected that they have relatively higher admittance than sample 1L, but lower than sample 3L, due to their similar resistance changes and stability under loading. The measured value for the sample 5L was ~447 µS.

**Figure 5:**
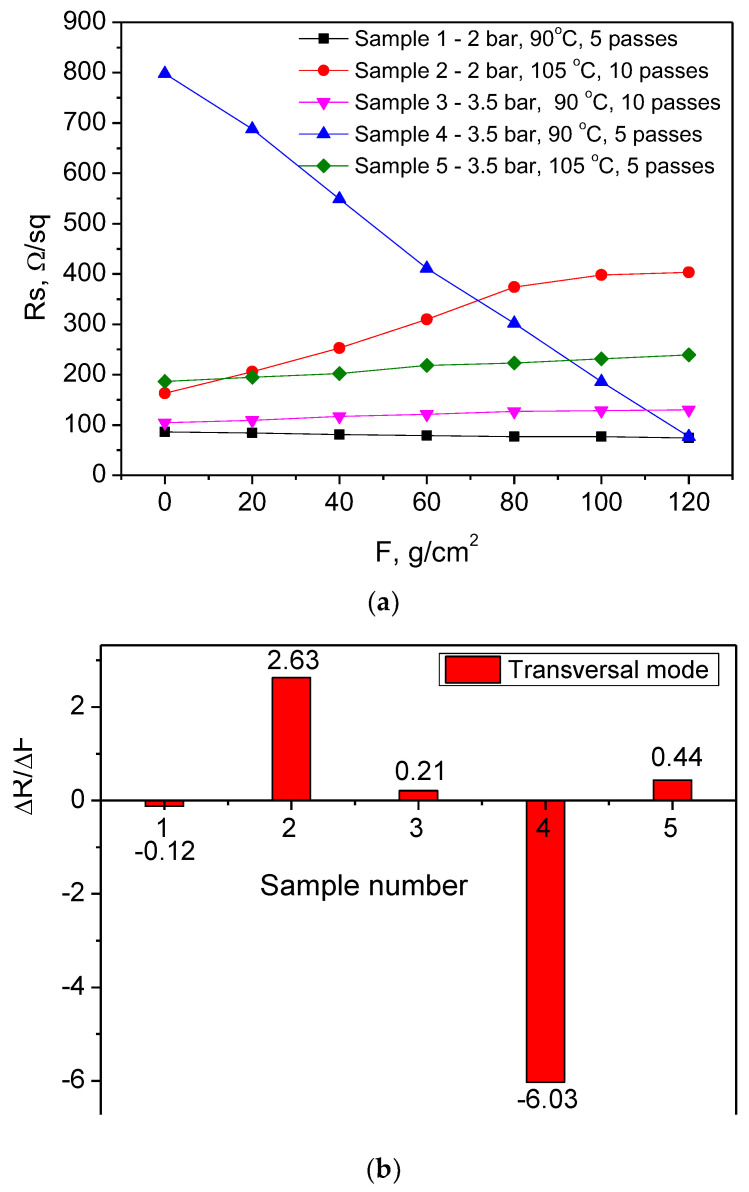
Sheet resistance variation (**a**) and sheet resistance degradation rate (**b**) for PEDOT:PSS/graphene films spray-coated at different conditions and subjected to transversal loading (denoted by T below).

Figure 5a,b show the results for the sheet resistance variation vs. applied force and degradation rates for all studied samples at transversal loading.

Again, sample 3T exhibits the most stable behavior, with its resistivity change of 0.21 Ω/sq per gram/cm^2^, which is similar to that under longitudinal loading (0.27 Ω/sq per gram/cm^2^). The next stable layer is sample 5T, which, under transverse loading, is much less sensitive (with ΔR of 0.44 Ω/sq per gram/cm^2^, whereas 2.03 Ω/sq per gram/cm^2^ was obtained under longitudinal loading). Sample 2T has a similar behavior to longitudinal loading (change in the sheet resistance here was 2.63 Ω/sq per gram/cm^2^ and in longitudinal loading 2.32 Ω/sq per gram/cm^2^). Sample 1T and sample 4T have an atypical behavior, as their resistance decreased instead of the expected increase. The change is negligible for sample 1T and significant for sample 4T, which has a very high initial resistance. After mechanical treatment, its value drops to be very suitable for electrode use. It was stable under longitudinal loading, but the high starting resistance was an obstacle for the previous loading case, in contrast to the present results.

**Figure 6:**
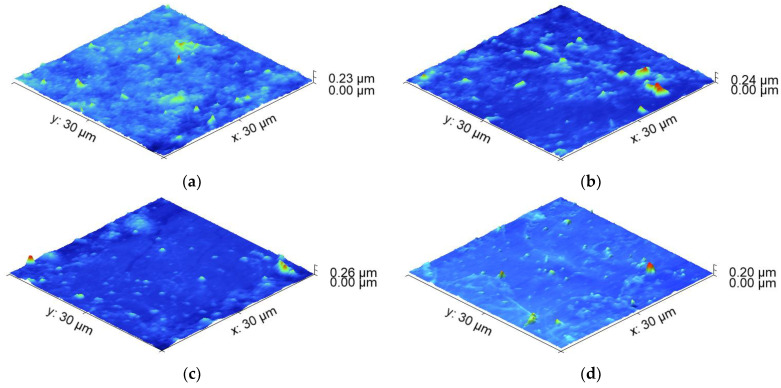
AFM 3D topography of the PEDOT:PSS/graphene coatings sprayed at different conditions and subjected to transversal loading for the samples with a thickness of (**a**) 3.1 µm; (**b**) 1.75 µm; (**c**) 2.5 µm; (**d**) 1.25 µm.

The interface between PEDOT:PSS and graphene is critical in determining the overall behavior of the composite. Mechanical loading may lead to changes in the interfacial interactions. The difference in the mechanical properties of the graphene and the polymer matrix can lead to stress and microparticle redistribution, impacting the electrical resistance of the composite. If the loading causes changes in the orientation or alignment of the graphene within the polymer matrix, this could lead to such atypical alterations in the electrical resistance. The PEDOT:PSS polymer matrix can exhibit complex behavior under mechanical loading, potentially changing its structure, orientation, or even localized deformations. These changes could also affect the conductivity of the composite material. We tried to provide insights into the mechanisms behind the observed resistance behavior with AFM microstructural and admittance electrical characterization following loading. AFM scans of the transversal loaded samples sprayed at different conditions were conducted, and the surface roughness was measured (Figure 6a–d).

Table 3 presents the relation between the resulting surface parameters of the transversal loaded films extracted from the AFM data and the spraying deposition conditions.

The results for the surface roughness of the coatings after transversal loading are in line to a great extent with the electrical characterization of the samples. Sample 5T, which is stable from slight sheet resistance variation, seems to undergo a negligible change in the surface roughness after loading (0.32 nm), comparable with the measurement error. As expected, sample 1T decreased its roughness after transversal loading by 3.2 nm, which is in agreement with the sheet resistance decrease and can be related to the redistribution of particles after this type of loading, taking a more favorable position for a more uniform and flat coating and the better interface between the particles in the composite, resulting in enhancement of the film’s conductivity. Indicative of the favorable redistribution in sample 1T is also the maximum peak height of the coating after longitudinal loading, which is higher (~224 nm) compared to the case of transversal loading (~95 nm). Additional investigation is required for samples 4T and 3T, in which surface roughness increased, although the expectations for negligible change for sample 3T and the negative direction of the surface variation for sample 4T show a negative value for the sheet resistance variation. A possible explanation is that changes in the interfacial properties between the composite material and the contacting electrodes may occur due to increased surface roughness, which can affect the transfer of charge carriers across the interface, influencing the overall sheet resistance measurements. It happens because the difference between the maximum peak height and the minimum pit depth is 81 nm for transversally loaded coating, in contrast to the longitudinal loading, where this difference is only 48 nm.

**Figure 7:**
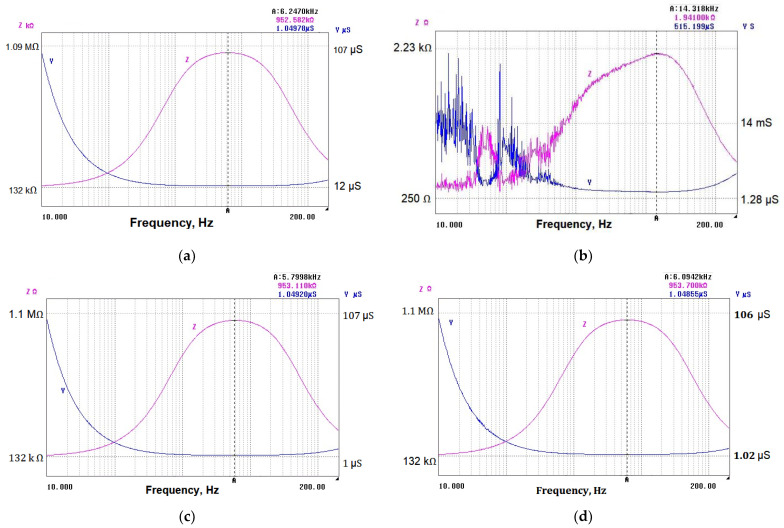
Admittance Y and impedance Z of samples with different thicknesses, subjected to transversal loading: (**a**) 3.1 µm; (**b**) 2.5 µm; (**c**) 1.75 µm; (**d**) 1.25 µm.

Admittance values extracted from the impedance characteristics for samples subjected to transversal loading (T-samples) are shown in Figure 7a–d. Considering the stability of sample 3T and its relatively low resistivity change, the achieved relatively high admittance is expected. Given the atypical behavior of samples 1T and 4T (negative change in the resistance), the admittance for these samples does not follow the expected pattern and requires further investigation. Sample 5T is characterized by a moderate admittance due to its decreased sensitivity under transversal loading.

**Figure 8:**
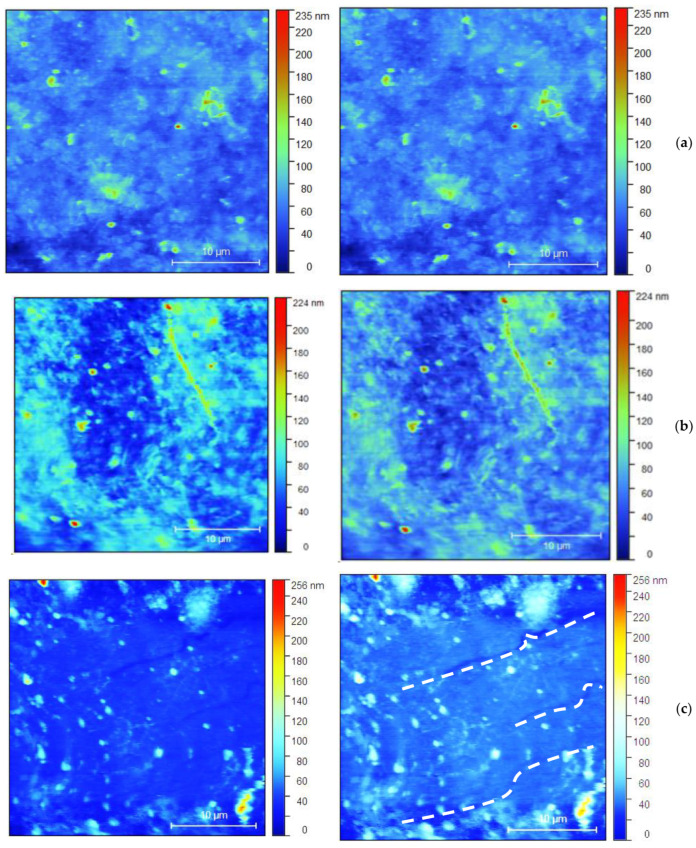

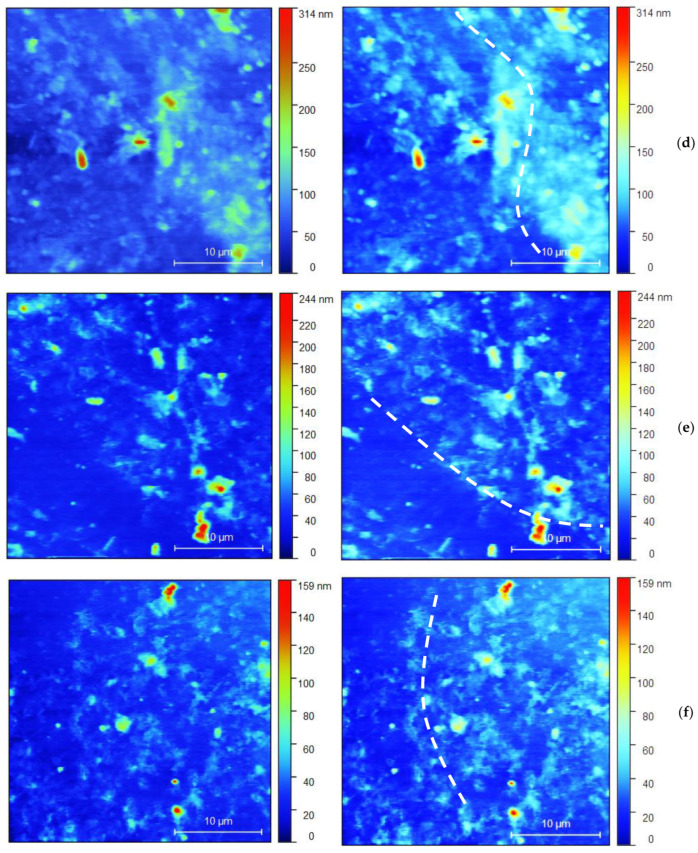

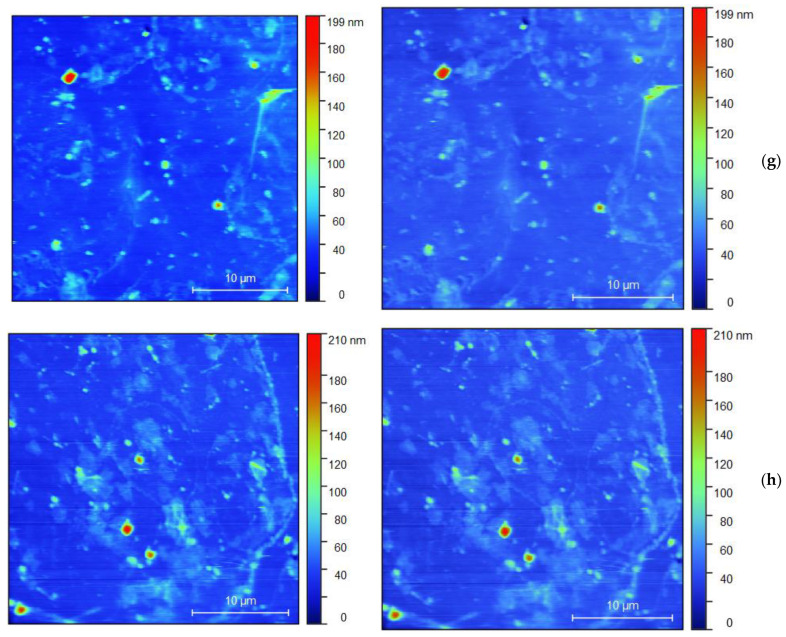
Optical microscopic images of all loaded samples (L and T denote the type of loading—longitudinal or transversal): (**a**) 1L; (**b**) 1T; (**c**) 3T; (**d**) 3L; (**e**) 4T; (**f**) 4L; (**g**) 5T; (**h**) 5L. In the **left** column, the samples before loading are presented and in the **right** one, those after the corresponding type of loading (L or T) are presented.

The microcracks and other damage to the coatings are well visible in the images from the optical micrograph (white dash lines), shown in Figure 8a–h. The right side color bar of the pictures indicates the height of the profile of each coating, similar to AFM topography.

As can be seen from Figure 8, on the sample 5T, two levels of damage over the surface coating layer are visible. First, the earliest is the initial bending edge or zone. The second stage is the appearance of surface cracks in the coating. It is observed that the initial bending edge that appears in the bending zones is marked by a white line. These edges are later transformed into surface cracks. Bending edges are wider than cracks, around 5 µm, while the crack width is less than 1 µm. Sample 5L exhibits a possible early-stage surface crack. For the samples 3T and 3L, surface cracks in the coating and possible initial bending edge without visible cracks are shown. Similarly, for samples 4T and 4L, possible initial bending edges with no visible cracks were obtained. For 1T and 1L, specific cracking is not observed.

**Figure 9:**
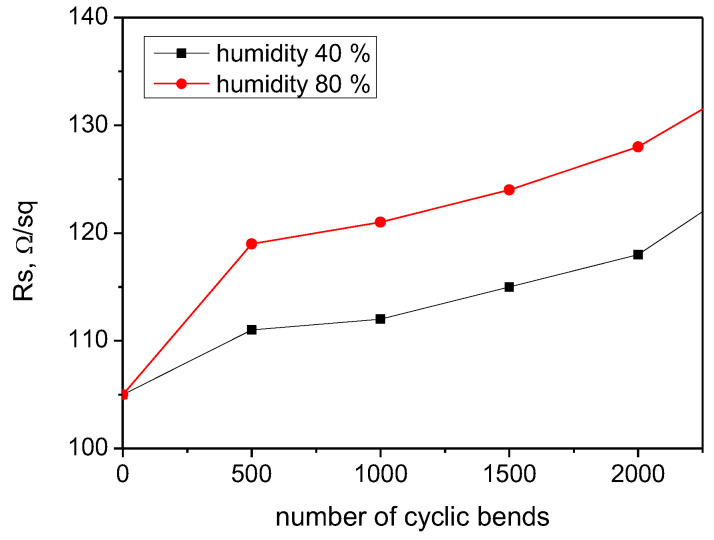
Sheet resistance variation in the PEDOT:PSS/graphene film prepared at 90 °C and 3.5 bar after multiple bends at 40% and 80% humidity.

For sample 3, identified as the most stable due to minimal change in the resistance without regard to the L or T mode of loading, a repeatable cycle bending test was conducted, and the resistance was measured after 500, 1000, 1500, and 2000 number of bends (Figure 9). The loading intensity was set to minimal (10 g/cm^2^) in order to avoid dual influence of the stimulus (strength of load and number of repeatable events) on the resistance variation. A gradually increasing resistance in a narrow range can be noted. The relative change of 12% of the sheet resistance was detected after 2000 bending cycles for a relative humidity of 40%, and the relative change in the resistance of 19% was measured after the same number of bendings at a relative humidity of 80%. The sheet resistance increase is due to the humidity adsorption to PEDOT:PSS, changing the charge transfer conditions between the graphene and PEDOT:PSS in the composite and not due to a violation of the surface integrity.

**Figure 10:**
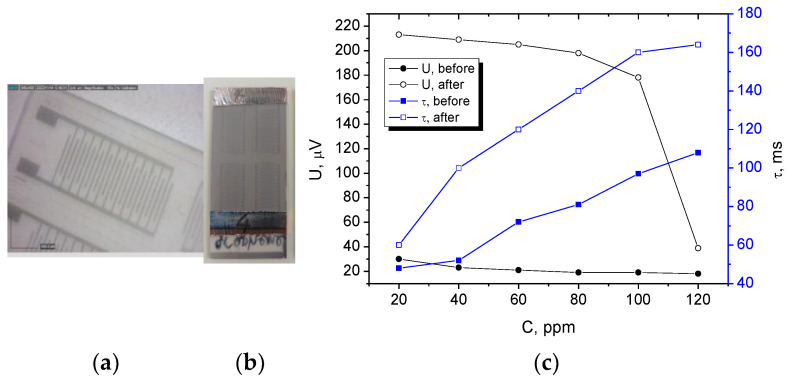
Fabricated sensing device with the proposed electrode coating: (**a**) closer view of the IDT patterned PEDOT:PSS/graphene electrode, (**b**) SAW sensor sample image, and (**c**) sensor response at different MgCl_2_ concentrations.

The applicability of the proposed electrode coating in biosensors was demonstrated by the fabrication of surface acoustic wave (SAW) sensors by using PEDOT:PSS/graphene as interdigitated electrode (IDT), coated on a piezoelectric polyvinylidene fluoride (PVDF) substrate and covered on the top with a Ti_3_C_2_ coating, is shown in Figure 10. The sinusoidal elastic wave was excited on the piezoelectric substrate with the help of a reference electrical AC signal applied to the IDT input transducer, and the conversion of acoustic waves to electrical signals was realized on the IDT output transducer. The Ti_3_C_2_ film is sensitive to MgCl_2_ from the sweat composition. MgCl_2_ sensing is a critical area of research with applications such as sweat analyzers for medical condition monitoring [28]. SAW sensors excel in providing quick response times, facilitating the real-time monitoring of sweat compound levels without significant delays. Increasing the MgCl_2_ concentration from 20 to 120 ppm increased the time delay between the output signal compared to the input reference signal *τ* with an average of 60 ms. An additional indication was the attenuation of the output voltage *U* with ~180 µV, comparing it to the magnitude of the input voltage after exposure to different concentrations of MgCl_2_.

## 4. Discussion

When considering mechanical loading, a mathematical explanation can be provided regarding stress and strain [29]. The stress (σ) can be defined as the force (F) applied over a specific cross-sectional area (A) of the sample:

σ = F/A(1)

This stress results in strain (ε) within the material. For a linear-elastic material, the strain can be expressed as the change in length (ΔL) per unit original length (L):

ε = ΔL/L.(2)

The relationship between stress and strain is described by the material’s modulus of elasticity or Young’s modulus (E), which measures the material’s stiffness. Mathematically, it is represented as:

E = σ/ε.(3)

When a material is subjected to mechanical stress, such as in the case of longitudinal or transversal loading, various internal processes influence its overall response. The probable processes to appear are deformation at the atomic scale, dislocation motion, or crack formation.

At the atomic level, the stress causes the atoms in the material to shift from their equilibrium positions slightly. This deformation results in the reorganization of atomic bonds and the creation of dislocations within the material [30]. This is the case for sample 1L (90 °C, 5 passes, 2 bar) and not the case of sample 3L (90 °C, 10 passes, 3.5 bar) when longitudinal loading is applied. The interaction between PEDOT:PSS and graphene at the interface plays a critical role. The degree of bonding and adhesion between the two components determines the stress transfer efficiency and affects the overall mechanical response [31]. Proper interfacial bonding can lead to enhanced mechanical properties, while weak bonding may result in premature failure [32]. Based on the data, the proper interface bonding is dominant for sample 3L (3T) in the case of longitudinal and also for transversal loading. Sample 5T (105 °C, 5 passes, 3.5 bar) is also in this category when transversal loading is applied. In response to stress, the material experiences strain. In the elastic range, the material deforms reversibly in response to the stress, and upon release of the stress, the material returns to its original shape. This is due to the reversible nature of atomic bond stretching and compression. It seems that the deposition conditions and loading modes for samples 1L when loaded longitudinally and 2T when loaded transversally result in a microstructure, which is not sufficiently reversible under stress release.

Regions of concentrated stress may lead to crack formation and ultimately fracture. This is especially relevant in materials where the coating or film integrity becomes compromised under high-stress conditions, explaining the connection between mechanical loading and the material’s behavior [33]. There is no such case among the studied samples, which is evidence that the composite with the graphene enhances the mechanical strength to a sufficient level avoiding irreversible damage such as microcracks, for example. Graphene, known for its exceptional mechanical properties, such as high tensile strength and stiffness, can reinforce the polymeric matrix, enhancing its overall mechanical behavior and stress resistance. When stress is applied, graphene can bear some of the load and distribute it throughout the polymeric matrix. This allows for a more uniform stress distribution and can minimize localized deformation. Its high aspect ratio and exceptional mechanical properties can impede crack propagation, enhancing the material’s overall durability and resistance to mechanical failure, even under prolonged loading conditions. Some deposition conditions and loading modes result in a non-homogeneous distribution of the graphene and PEDOT:PSS, resulting in a sharp degradation rate of the resistance of these samples, for example, 1L. The number of passes, pressure, and temperature during the coating process directly impact the film’s thickness, uniformity, and internal structure [34]. Variations in these conditions can lead to differences in the arrangement of atoms and molecules at the atomic scale within the composite material. For instance, a higher number of passes or increased pressure lead to a denser packing of the materials within the composite, affecting atomic-scale interactions. Higher pressure and temperature can influence the degree of crystallinity, grain size, and orientation of the graphene component, directly affecting the material’s susceptibility to dislocation movement and subsequent plastic deformation when subjected to mechanical stress [35]. Optimizing the deposition parameters can lead to a greater alignment of graphene flakes within the polymeric matrix, enhancing load transfer mechanisms and thereby improving mechanical properties under stress.

The behavior of the samples under transversal mechanical loading is likely to exhibit different effects compared to longitudinal loading due to the anisotropic nature of the composite material and the unique responses to various stress orientations [36].

The composite material consisting of PEDOT:PSS and graphene is inherently anisotropic due to the orientation of the graphene flakes within the polymer matrix. As a result, transverse loading will subject the material to stress across the plane, potentially causing different deformation and fracture behavior compared to longitudinal loading. Transverse loading introduces shear stress and deformation across the interfaces between the polymer and the graphene sheets. This may influence interfacial bonding differently, potentially leading to delamination or sliding at the interface. According to the measured data, this is the case for sample 2 (105 °C, 10 passes, 2 bar), which was eliminated for further study because of this reason.

Transverse loading can induce distinct microstructural changes within the material. The orientation and alignment of the graphene flakes and their interaction with the polymer matrix will respond differently to transverse stress, potentially leading to unique deformation modes and resistance change mechanisms. This should be the case for the negative variation in the resistance (or improved electrical conductivity) of the transversally loaded samples 1T and 4T.

Regarding the comparison of the performance of the proposed electrode coating to other works (Table 4), most of the papers investigate conductive composites for stain sensors and evaluate the resistance change at different strains, strain rates and strain cycles. Some of the researchers use PEDOT:PSS as a base for the composites preparation, and the results are in the same range as those proposed in this work, i.e., resistance change up to 10, according to the geometry and mechanical stimuli. For example, the change in the electrical resistance of the conductive polymer fibers PEDOT:PSS/PBP (polyethylene-block-poly (ethylene glycol)) at stretching of 30%, is approximately four-fold as compared to its initial value. At multiple stretching/unstretching cycles, it reaches seven-fold [37]. The resistance-strain relations of PANI:PAMPSA (polyanilin:poly(2-acrylamido-2-methyl-1-propanesulfonic) at strain of 100% under different strain rates between 2.5 and 2500%/min show resistance change between 10- and 20-fold [38]. The super-aligned carbon nanotube interlayer (SACNT) inserted between the platinum and PDMS as a secondary conductive network bridged gaps in the platinum layer, preventing abrupt increases in resistance during stretching and thereby maintaining linearity up to 100% strain. However, the resistance change in the multilayer system is approximately 16-fold at strain lower than 5% [39]. Therefore, comparing the results with those reported in the literature, PEDOT:PSS/graphene has not been extensively investigated in different mechanical loading modes. Both the L and T modes of loading result in similar and even smaller sensitivity to loading intensity with respect to the resistance change. The reason is the reinforcing properties of the graphene. When graphene is incorporated into PEDOT:PSS composites, it enhances the overall mechanical behavior of the composite due to its high aspect ratio and strong interfacial interactions with the polymer matrix. This improves mechanical stability, making the material more resistant to deformation and structural damage under stress. The advantage of the proposed technology in our study is the atomization of the solution, making possible precise control of the film growth at the molecular level and leading to uniform, smooth coating formation (as is proved by the AFM). This particle arrangement results in a lack of stress points and relatively poor cracking compared to other conductive polymer-based composites.

## 5. Conclusions

This study investigates the behavior of PEDOT:PSS/graphene films when subjected to longitudinal and transversal loading conditions. It was found that the samples sprayed at 3.5 bar, 10 passes, and 90 °C exhibited the most stable resistance under longitudinal loading, with a resistance change of 0.27 Ω/sq per gram/cm^2^ and an initial resistance of approximately 100 Ω/sq, making it suitable to serve as an electrode. Samples sprayed at the same high temperature exhibited similar low-resistance changes, no matter the different pressures. Samples sprayed at 105 °C demonstrated stability under both loading conditions, showing minimal resistance change regardless of the spraying pressure. The consistent behavior of these samples under both longitudinal and transversal loading indicates that the graphene component effectively reinforces the polymer matrix, enhancing the material’s mechanical resilience. The temperature of spray deposition can influence the distribution and alignment of graphene within the polymer matrix. Optimal temperature settings during deposition can promote better dispersion of graphene particles, leading to a more uniform reinforcement throughout the composite. This uniform distribution contributes to enhanced mechanical properties and resilience against mechanical stress. After 2000 cyclic bends, the initial sheet resistance increased by less than 20%, even in highly moist environments. The applicability of the proposed electrode coating in the biosensing structure was successfully demonstrated by the detection of a sweat component, MgCl_2_, in the range of 20–120 ppm with a SAW-type of sensor using patterned IDT electrodes of PEDOT:PSS/graphene.

The composite’s electrical response is closely connected to its mechanical behavior. Changes in the composite’s microstructure under stress alter its electrical conductance, influencing its suitability for use as an electrode or in other electronic applications. The composite nature of the material, integrating PEDOT:PSS and graphene, alters its mechanical response to stress. The presence of graphene can not only reinforce the mechanical properties but can also influence the load transfer, interfacial bonding, and microstructural changes, all of which are crucial in determining its mechanical and electrical behavior under longitudinal and transversal loading conditions.

In conclusion, for the series of samples subjected to longitudinal loading, the interplay between the deposition conditions and the atomic-scale characteristics, load transfer, interfacial bonding, and microstructural changes highlights the critical role of precise and controlled fabrication processes in tailoring the mechanical and electrical properties of the composite material. By optimizing deposition conditions, it is possible to modulate the material’s internal structure and, in turn, its response to external stress, laying the foundation for advanced engineering of materials with tailored mechanical and electrical properties.

In summary, for the series of samples subjected to transversal loading, given the anisotropic nature of the composite material and the complex interplay between the polymer matrix and graphene components, the samples are expected to behave differently under this type of mechanical loading compared to longitudinal loading. The unique responses to transverse stress are likely to manifest as distinct failure mechanisms, deformation modes, and changes in electrical behavior, showing the need for comprehensive characterization under various loading conditions to fully understand the material’s mechanical and electrical properties. Further improvement can be achieved by adjusting the graphene concentration in the composite, as finding the optimal loading percentage can enhance the performance. The PEDOT:PSS matrix can be subjected to crosslinking or copolymerization. Also, the implementation of post-treatment processes like chemical doping can help improve the crystallinity and, therefore, mechanical stability and conductivity of the composite.

Future work will study parasitic capacitances formed at the junction between the PEDOT:PSS film that could be used as an electrode and a functional layer. In the present study, the impedance value reflects the reactive and active reactance. In the case of conducting polymers, the reactive resistance (or parasitic capacitance) can be related to areas of heaped material that tend to form an uncompensated charge upon contact with another layer. This results in a depleted junction region and creates a parasitic capacitance that is strongly affected by the voltage and which contributes to the reactive components, affecting the frequency response of a capacitive type of sensors, for example. Another direction for future exploring is the electroanalytical performance of the films after exposure to different mechanical stresses. There is a probability of introducing changes in the material’s structure, morphology, and electrical properties due to the bending, which may influence its electrochemical behavior. For instance, bending could alter the surface area available for electrochemical reactions, affect the transport of ions or electrons within the material, or induce mechanical damage that hinders performance. Such study and analysis can provide critical insights into the stability, durability, and performance of the films under real-life conditions, allowing for a more comprehensive understanding of their electrochemical behavior.

## Figures and Tables

**Table 1:** Fabrication conditions of spray-deposited film from PEDOT: PSS/graphene.

Sample No #	T, °C	No # Passes	Pressure, Bar	Thickness, µm	Rs, Ω/sq
1	90	5	2	3.1	86.2
2	105	10	2	3	196
3	90	10	3.5	2.5	104.4
4	90	5	3.5	1.75	800.3
5	105	5	3.5	1.25	198

**Table 2:** Surface roughness of the AFM scanned PEDOT:PSS/graphene samples sprayed at different sample conditions before and after being subjected to longitudinal loading. The index L denotes longitudinal mode of loading.

Sample N#	RMS Roughness Initially—S_q_, nm	RMS Roughness after Load S_q_, nm	Mean Roughness after Load S_a_, nm
1L	16.7	36.41	21.78
3L	10.03	12.81	9.79
4L	7.71	12.05	8.60
5L	5.95	10.34	7.49

**Table 3:** Surface roughness of the AFM scanned PEDOT:PSS/graphene samples sprayed at different sample conditions before and after being subjected to transversal loading. The index T denotes transversal mode.

Sample N#	RMS Roughness Initially—S_q_, nm	RMS Roughness after Load S_q_, nm	Mean Roughness after Load S_a_, nm
1T	16.7	13.52	10.19
3T	10.03	15.35	11.05
4T	7.71	18.01	13.07
5T	5.95	5.63	4.02

**Table 4:** Comparison between the performance of the proposed PEDOT:PSS/graphene electrode and recently reported PEDOT:PSS-based composites.

Composite Type	Substrate Type	Deposition Method	RMS Surface Roughness after Loading	Mechanical Loading	Sheet Resistance	References
PEDOT:PSS/Carbon Nanotubes (CNTs)	Polyamide taffeta fabric	Ink-jet printing	154.7 nm	Up to 1000 cycles with a bending radius of 6 mm	Variation of 940 Ω/sq	[40]
PEDOT:PSS/GNPs (Graphene Nanoparticles)	Cotton fabrics	Spray deposition	N/A	Up to 1000 bending cycles at bend folding of 180°	Variation of 300 Ω/sq	[41]
PEDOT:PSS/Carbon Nanofibers (CNFs)	Polydimethylsiloxane (PDMS)	Vacuum-assisted filtration	2.48 nm	80 stretching cycles up to 40%	Variation of 55 Ω/sq	[42]
PEDOT:PSS/Silver Nanowires (AgNWs)	Kapton	Electrospinning	N/A	Up to 1000 bending cycles	Variation of 7 Ω/sq	[43]
PEDOT:PSS/graphene	Polyethylene terephthalate (PET)	Spray deposition	10.3 nm for longitudinal loading and 5.6 nm for transversal loading	Up to 2000 bending cycles	Variation of 22 Ω/sq (@ 40% humidity) and 30 Ω/sq (@ 80% humidity)	This work

## Data Availability

Dataset available on request from the authors.
